# Supplementary material for: Detailed molecular and epigenetic characterization of the pig IPEC-J2 and chicken SL-29 cell lines
Source: iScience. 2023 Feb 20;26(3):106252. doi: 10.1016/j.isci.2023.106252 (PMC10018572; doi:10.1016/j.isci.2023.106252)
Supplement: Data S1. Complete homer output for identified motifs in Pig IPECJ-2, related to Table 2 — Homer motif analysis results for histone modifications H3K4me1, H3K4me3, H3K27ac, and enhancer elements of pig IPECJ2 cell line. P-values >1e-10 are possible false positives. Within each folder (e.g. peak_files_CTCF) are the html files showing the identified motifs when using homer (e.g. homerResults.html). [file mmc2.zip › S5/Pig_IPECJ_2/peak_files_H3K27ac/homerResults/motif17.similar.html]

motif17

## Information for motif17

G
C
A
T
G
C
T
A
T
A
C
G
A
C
G
T
A
G
C
T
G
A
T
C
T
A
C
G
T
C
A
G
T
A
C
G
T
A
G
C
  
Reverse Opposite:  

A
T
C
G
A
T
G
C
A
G
T
C
A
T
G
C
C
T
A
G
T
C
G
A
T
G
C
A
A
T
G
C
C
G
A
T
C
G
T
A
  

|  |  |
| --- | --- |
| p-value: | 1e-49 |
| log p-value: | -1.144e+02 |
| Information Content per bp: | 1.462 |
| Number of Target Sequences with motif | 5788.0 |
| Percentage of Target Sequences with motif | 11.01% |
| Number of Background Sequences with motif | 4711.2 |
| Percentage of Background Sequences with motif | 9.09% |
| Average Position of motif in Targets | 149.9 +/- 83.7bp |
| Average Position of motif in Background | 149.4 +/- 92.2bp |
| Strand Bias (log2 ratio + to - strand density) | 0.0 |
| Multiplicity (# of sites on avg that occur together) | 1.06 |
| Motif File: | file (matrix) reverse opposite |

### Similar de novo motifs found

|  |  |  |  |  |  |  |  |
| --- | --- | --- | --- | --- | --- | --- | --- |
| Rank | Match Score | Redundant Motif | P-value | log P-value | % of Targets | % of Background | Motif file |
| 1 | 0.677 | T C G A T C A G C G A T G A T C A G T C A T C G T A G C A T C G | 1e-47 | -110.124834 | 24.70% | 22.02% | motif file (matrix) |
| 2 | 0.612 | C A G T T C G A A T C G C A G T A T G C G T A C A T G C C T A G | 1e-42 | -96.814891 | 6.82% | 5.42% | motif file (matrix) |
| 3 | 0.617 | A G C T G A C T C G T A A C T G T C G A A G C T G T A C G C T A C T A G C T A G T G A C G A T C G A C T | 1e-22 | -52.716404 | 0.06% | 0.00% | motif file (matrix) |
